# Lung cancer coexisting with *Papiliotrema flavescens* infection diagnosed by next-generation sequencing: a case report

**DOI:** 10.1186/s12879-022-07591-0

**Published:** 2022-08-09

**Authors:** Siang Zhang, Liangzhe Wang, Qianyu Han, An Sun, Xiaogang Liu, Lei Xue

**Affiliations:** 1 Department of Thoracic Surgery, Changzheng Hospital, Naval Medical University, 415 Fengyang Road, Shanghai, 200003 China; 2 Department of Pathology, Changzheng Hospital, Naval Medical University, Shanghai, China; 3 Department of Oncology, Medical Center, Naval Medical University, Shanghai, China; 4 Department of Radiology, Changzheng Hospital, Naval Medical University, Shanghai, China; 5 Department of Dermatology, Changzheng Hospital, Naval Medical University, 415 Fengyang Road, Shanghai, 200003 China

**Keywords:** Case report, *Papiliotrema flavescens*, Lung cancer, Adenocarcinoma, Pulmonary fungal infection, Next-generation sequencing

## Abstract

**Background:**

*Papiliotrema flavescens* is a rare environmental yeast, which has been isolated from air, trees, kernels of wheat and corn, fermenting soya sauce, and cerebrospinal fluid of patient with AIDS. Additionally, it is also reported to cause subcutaneous infection in a dog. In this case, we describe primary lung adenocarcinoma coexisting with *Papiliotrema flavescens* infection in a female patient diagnosed by next-generation sequencing (NGS) technique, which is the first such reported case.

**Case presentation:**

The patient was a 52-year-old female with recurrent cough for 3 months. Chest CT examination revealed a ground glass nodule of 17 * 23 * 18 mm in the right upper lung, and 3 new pulmonary nodules appeared around it 2 months later. The patient underwent right upper lobe lobectomy and pathology confirmed that the primary 2-cm-lesion in the right upper lobe was invasive lung adenocarcinoma, and two of the three surrounding lung nodules were pathologically suggestive of pulmonary fungal infection (not known in specific fungal types). Hence, the patient received empirical anti-fungal treatments with fluconazole 400 mg/day for a week and follow-up CT scanning showed no tumor progression and no relapse of fungal infection. The specific pathogen was eventually identified as *Papiliotrema flavescens* by the next-generation sequencing of pathogen.

**Discussion and conclusion:**

We first reported that lung cancer coexisting with *Papiliotrema flavescens* infection in a female patient. The diagnosis of lung cancer with typical CT imaging features is relatively simple, while the diagnosis of lung cancer coexisting with rare fungal infection is challenging. NGS technique is an effective supplementary technique for clinical diagnosis of bacterial or fungal infectious diseases, enabling precise clinical decision-making and appropriate treatment. In this case, the lung cancer may result in a degree of immune suppression, at least locally, resulting in the formation of pulmonary fungal nodular lesions around the tumor.

## Background

*Papiliotrema flavescens*, formerly known as *Cryptococcus flavescens* and previously considered to be a synonym of *Cryptococcus laurentii*, was identified as a separate species based on sequences of the D1/D2 region of 26S rDNA and internal transcribed spacer (ITS) regions [1–5]. It has been isolated from air, trees, kernels of wheat and corn, fermenting soya sauce and cerebrospinal fluid of a patient with AIDS [3, 6]. Besides, it is also reported to cause subcutaneous infection in a dog, indicating that *Papiliotrema flavescens* is a potential canine pathogen [7]. As we have previously known, most of these “non-pathogenic to human” fungus often cause opportunistic infections in immunocompromised patients. However, here we report primary lung adenocarcinoma coexisting with *Papiliotrema flavescens* infection in a female patient diagnosed by next-generation sequencing (NGS) technique. To the best of our knowledge, this is the first such reported case.

## Case presentation

The patient was a 52-year-old female with recurrent cough for 3 months. Chest CT examination at local hospital revealed a ground glass nodule of 17 * 23 * 18 mm in the right upper lung. The patient felt no chest tightness or chest pain, and had no other symptoms such as fever, fatigue or night sweats. The patient had no known cause of immunosuppression, no known drug allergies, and no smoking or alcohol consumption history. The patient had been living in Shanxi Province, China and had no recent travel history.

**Fig. 1:**
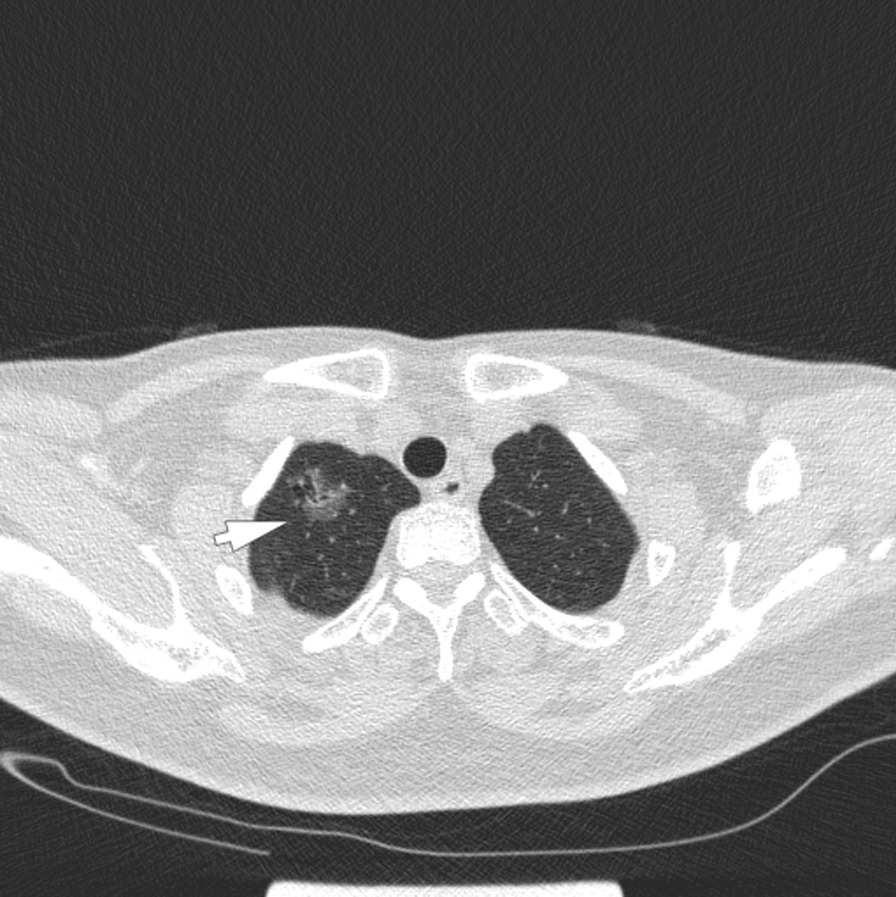
Chest CT scan showed a grinded glass nodule (white arrow) in the right upper lobe

**Fig. 2:**
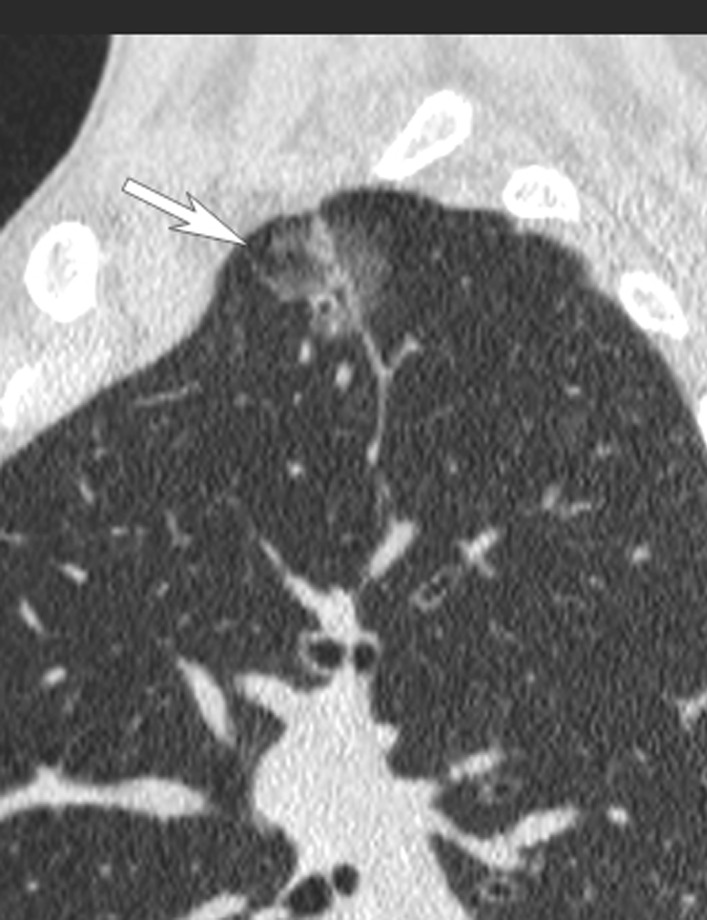
Main lesion showed in CT sagital reconstruction

**Fig. 3:**
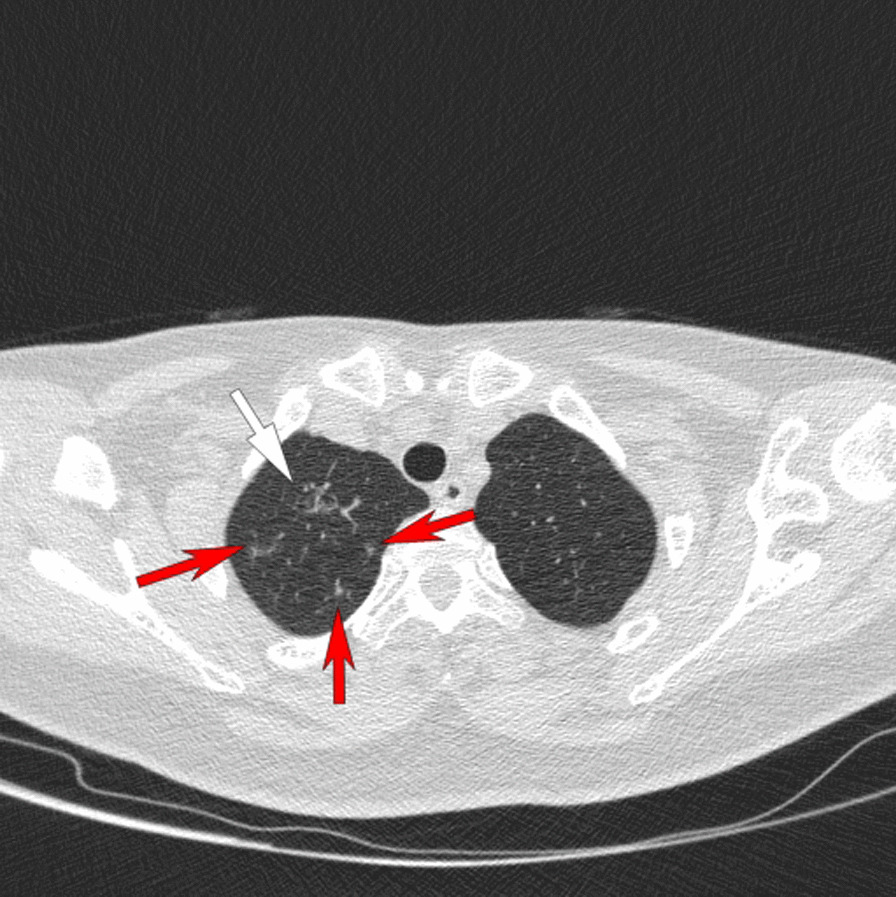
CT showed 3 smaller lung nodules (red arrows) were found surrounding the lower polar of the main lesion (white arrow)

The follow-up chest CT scanning in our hospital 2 months later (June, 2020) showed significant change in the previous lung nodule. In addition, 3 new emerged lung nodules (4 mm, 5 mm and 6 mm in distance respectively) were found around it (Figs. 1, 2 and 3). Therefore, the patient was admitted to the hospital for surgical treatment. Routine preoperative laboratory tests including complete blood count, blood glucose, aspartate aminotransferase, alanine aminotransferase, creatinine, hepatitis B virus surface antigen (HBsAg) test, anti-hepatitis C virus (anti-HCV) test, HIV test and tolulized red unheated serum test (TRUST) were normal. Since CT showed that the primary lesion in the right upper lobe was highly suspicious of malignancy, and considering that all lung nodules were located in the same lobe, we first performed wedge resection of right upper lobe via VATs including all the four lesions, and the intraoperative frozen section suggested lung invasive adenocarcinoma (the main lesion). Therefore, the final surgical procedure was converted to right upper lobe lobectomy and systematic lymph node dissection via VATs as a standard surgical treatment for lung cancer. The patient was discharged successfully 7 days after operation.

**Fig. 4:**
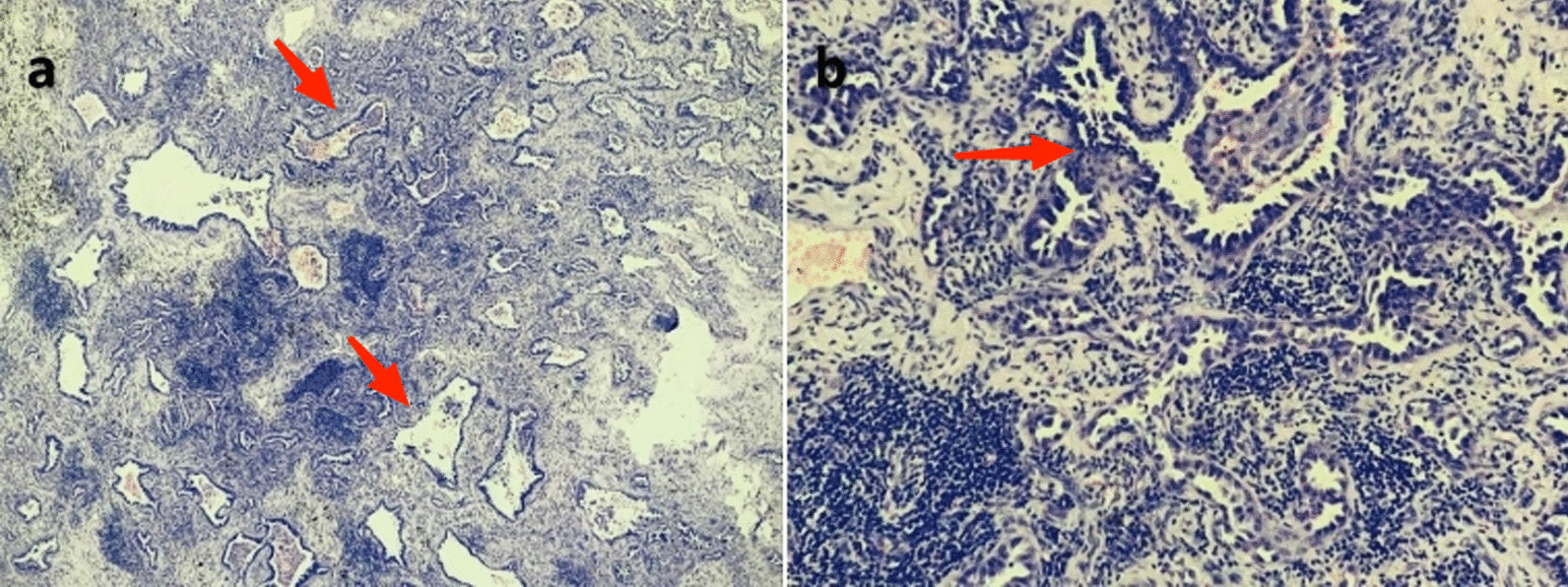
HE stain (**a** ×40, **b **×200) showed a typical acinar predominant adenocarcinoma in the primary lesion. Tumor consisted of primarily of acinar invasion with small area of lepidic pattern growth (red arrows)

**Fig. 5:**
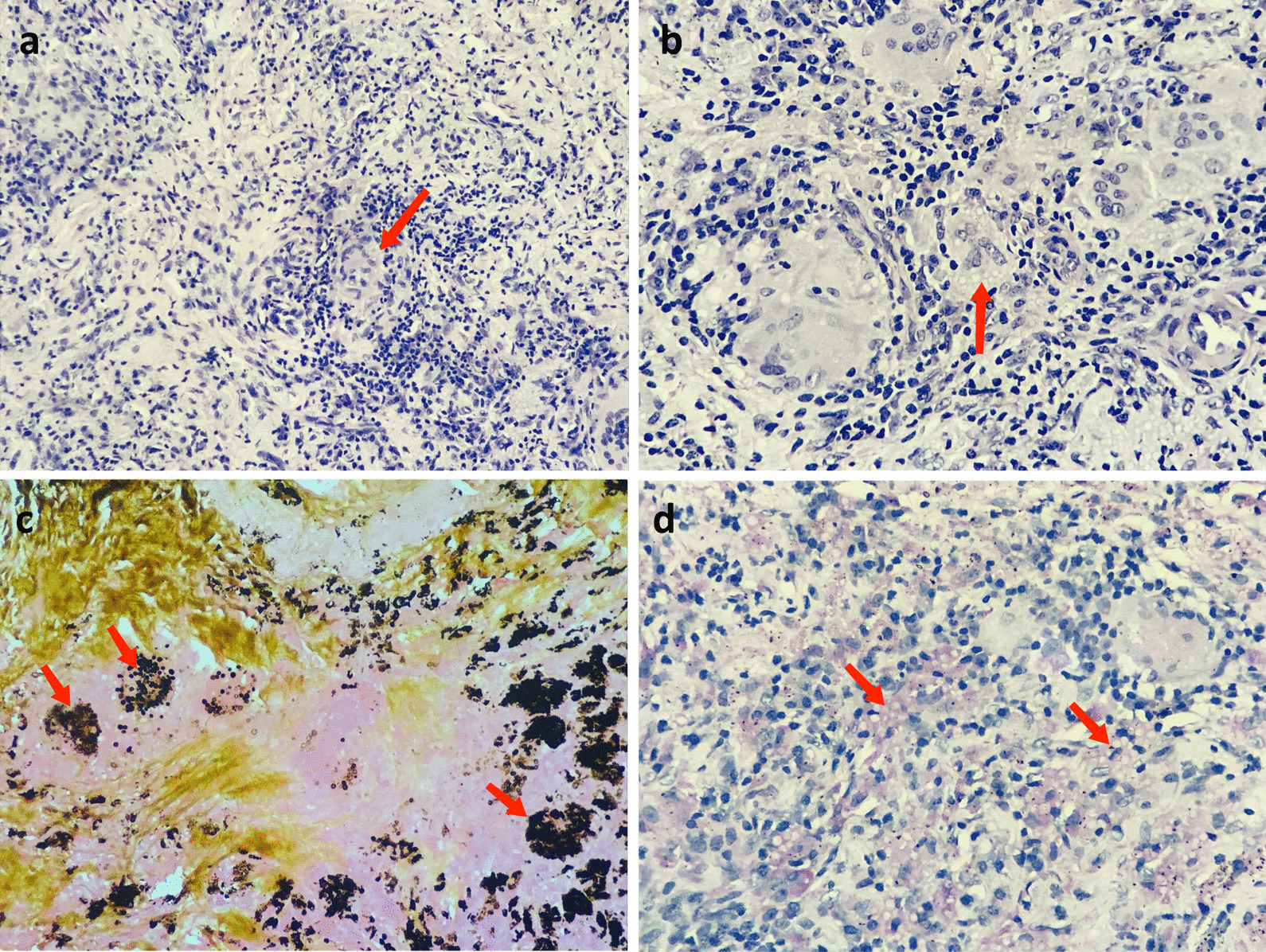
HE stain (**a **×100, **b** ×400) showed a large number of fungal spores in the granuloma (**a**, **b**), which can be confirmed in both Grocott’s Methenamine Silver (GMS) stain (**c**) and Periodic Acid-Schiff (PAS) stain (**d**) (red arrows)

Postoperative paraffin pathology confirmed that the primary 2-cm-lesion in the right upper lobe was invasive lung adenocarcinoma (Fig. 4), and there were no metastases in the lymph nodes at any station. Intriguingly, however, two of the three surrounding lung nodules were pathologically suggestive of pulmonary fungal infection (not known in specific fungal types). Therefore, the patient was contacted and informed. After readmission 1 month postoperatively (July 28, 2020), the patient underwent a series of mycological examinations. The serologic tests including latex agglutination test, (1,3)-beta-d-glucan (bDG) test and galactomannan (GM) test all yielded negative results. And blood cultures for fungus and bacteria were negative either. The sputum cultures were positive for *Candida albicans*, but because the sample was from the oral cavity, the results were not entirely reliable. In terms of histopathological evaluation of the postoperative paraffin, many fungal spores could be found in granulomatous with a careful observation under the high magnification in HE stain (Fig. 5a, b), which was double confirmed in both Grocott’s Methenamine Silver (GMS) stain (Fig. 5c) and Periodic Acid-Schiff (PAS) stain (Fig. 5d). Cryptococcal infection was then suspected based on the morphological findings in histopathology, so a course of empirical anti-fungal therapy with voriconazole (400 mg/day for a week) was applied. The patient’s final diagnosis was not revealed until the result of next-generation sequencing of pathogen (PMseq^®^-DNA, BGI) came out.

Next-generation sequencing (NGS) is an unbiased technique that can theoretically detect all pathogens in a sample. After DNA extraction from paraffin samples and library construction, nucleic acids were sequenced to identify the suspected pathogen present in the sample, including 6350 bacterial species (including 133 Mycobacteria and 122 Mycoplasma/Chlamydia/Rickettsia), 1798 DNA viruses, 1064 fungi and 234 parasites with known genome sequences. The NGS results are as follows: The number of raw reads was 30,261,384. The sequence reads of the fungi identified corresponding to *Papiliotrema flavescens* were 34, which matched previous histopathological finding. The details of NGS results are presented in Table 1. The final diagnosis was lung cancer coexisting with *Papiliotrema flavescens* infection. Given that no new lesions were found on follow-up chest CT, the patient discontinued anti-fungal therapy (July 29, 2020–August 5, 2020) and was discharged from the hospital.

**Table 1:** Number of reads aligning to fungi sequences

Species	Coverage	Coverage rate (%)	Reads
*Papiliotrema_flavescens*	1634/22797593	0.0072	34
*Melampsora_pinitorqua*	1819/33400632	0.0054	16
*Plasmopara_obducens*	412/200436746	0.0002	4
*Plasmopara_halstedii*	582/342262434	0.0002	3
*Cronartium_quercuum*	174/21759774	0.0008	5
*Aspergillus_fischeri*	215/31766197	0.0007	2
*Aspergillus_sydowii*	86/34353674	0.0003	2
*Rhodotorula_mucilaginosa*	172/20219435	0.0009	4
*Phoma_herbarum*	129/38514728	0.0003	3
*Yarrowia_lipolytica*	129/20549933	0.0006	3
*Cyphellophora_europaea*	86/28720667	0.0003	2
*Ophiognomonia_clavigignenti-juglandacearum*	149/52567057	0.0003	2
*Purpureocillium_lilacinum*	86/38218893	0.0002	2
*Balansia_obtecta*	43/30169048	0.0001	1
*Cadophora_malorum*	43/47805856	0.0001	1
*Cystobasidium_pallidum*	43/21261313	0.0002	1
*Epichloe_sylvatica*	129/36463153	0.0004	1
*Epichloe_brachyelytri*	43/44283290	0.0001	0
*Komagataella_pastoris*	43/9482831	0.0005	0
*Malassezia_restricta*	43/7369717	0.0006	1
*Nigrograna_mackinnonii*	43/51714876	0.0001	1
*Ochroconis_constricta*	43/34439787	0.0001	1
*Trichophyton_interdigitale*	126/22623371	0.0006	1

## Discussion and conclusion

*Papiliotrema flavescens* was reported caused subcutaneous infection in a dog [7] and had been isolated from the cerebrospinal fluid of a patient with AIDS [6]. This is the first case report describing *Papiliotrema flavescens* infection in a patient with no known cause of immunosuppression, additionally coexisting with lung cancer.

Since CT feature of pulmonary fungal infection can present as solitary or multiple nodules, it is not easy to distinguish from lung cancer. Furthermore, in this case, the primary lesion was highly suspicious of malignancy from CT Imaging, coupled with the later appearance of the *Papiliotrema flavescens* nodules located close to the primary tumor and morphologically similar to the malignant lung nodules, which made it more likely for clinicians to misdiagnose.

In this case, all the results of serologic tests, blood and sputum cultures were negative, histopathology could not confirm the fungus specie either, and the eventual diagnosis could not be drawn without NGS technique. NGS is a new technique that is increasingly used for the clinical diagnosis of bacterial or fungal infection in infectious diseases. Theoretically, assuming there were sufficiently long reads and a complete genomic reference database, almost all pathogens can be uniquely identified based on their specific nucleic acid sequences. It is especially suitable for challenging case that cannot be solved by conventional diagnosis methods.

Lung cancer coexisting with pulmonary fungal infection is rare, and its correlation between them has been controversial and inconclusive. Robinson et al. [8] believed that tumors can suppress immunity and increase the body’s susceptibility to fungal infections. However, Harada et al. [9] considered that the co-infection is simply a coincidence. In this case, the fungus infection nodules occurred after the cancer, and were satellite-shaped, closely surrounding the primary tumor and confined to the same lung lobe. As we have previously known, most of these “non-pathogenic to human” fungi often cause opportunistic infections in immunocompromised patients. We might not quite rigorously infer that the tumor resulted in a degree of immune suppression, at least locally, and thus the formation of pulmonary fungal nodular lesions around the tumor.

In summary, we first reported that lung cancer coexisting with *Papiliotrema flavescens* infection in a female patient. The diagnosis of lung cancer with typical CT imaging characteristics is relatively simple, while the diagnosis of lung cancer coexisting with rare fungal infections is challenging. NGS technique is an effective supplementary technique for the clinical diagnosis of bacterial or fungal infectious diseases, enabling precise clinical decision-making and appropriate treatment. In this case, lung cancer may result in a degree of immune suppression, at least locally, resulting in the formation of pulmonary fungal nodular lesions around the tumor.

## Data Availability

Not applicable.

## Abbreviations

NGS: Next-generation sequencing
bDG test: (1,3)-Beta-d-glucan test
GM test: Galactomannan test
GMS: Grocott’s Methenamine Silver
PAS: Periodic Acid-Schiff
